# Complete chloroplast genome and evolutionary analysis of *Acer paihengii* (Sapindales:Aceraceae)

**DOI:** 10.1080/23802359.2022.2119102

**Published:** 2022-09-07

**Authors:** Yiping Liu, Yunru Zhai, Dan He, Hongli Liu, Man Zhang, Dezheng Kong

**Affiliations:** a College of Landscape Architecture and Art, Henan Agricultural University, Henan, China; b Henan High-quality Flower and Vegetable Seedling Engineering Technology Center, Henan, China

**Keywords:** *Acer paihengii*, chloroplast genome, phylogenetic analysis

## Abstract

In this study, the complete chloroplast genome of *Acer paihengii*, a tree species native to China, was sequenced and assembled through second-generation sequencing. The complete chloroplast genome of *A. paihengii* is 155,967 bp in length with a typical quadripartite structure, encompassing 130 genes including 85 protein-coding genes, 37 tRNA genes, and 8 rRNA genes. Phylogenetic analysis of 22 related species indicated that *A. paihengii* was more closely related to *Acer coriaceifolium* and *Acer sino-oblongum*.

*Acer paihengii* is a unique deciduous tree species with high ornamental value in China. It is mainly distributed in Yunnan Province, China, where it is a provincial key protected wild plant species (Zhou 2010; Qin et al. 2017). Most studies on Aceraceae plants in China mainly focus on genetic breeding, introduction and domestication, cultivation techniques, economic uses, and landscape ecological applications, among other aspects. However, few studies have focused on *A. paihengii*. The research involved in this species mainly focused on resource investigation and biodiversity.

The chloroplast (cp) genome is highly conserved among plants due to its semi-autonomous and maternal inheritance characteristics, and can thus provide important molecular data onto the characterization of plant systematic evolution and biogeography research (Gao et al. 2020; Yang et al. 2020). Here, the complete chloroplast genome of *A. paihengii* was assembled, annotated, and phylogenetically analyzed, thus providing crucial insights into the evolutionary relationship between *A. paihengii* and other members of the *Acer* genus such as *Acer miaotaiense* (Zhang et al. 2016), *Acer buergerianum* (Xu et al. 2017), *Acer saccharum* (Deng et al. 2019), *Acer truncatum* (Chen et al. 2019), and *Acer tataricum* subsp. *ginnala* (Yang et al. 2020).

*Acer paihengii* Fang was first mentioned in Act. Phytotax. Sin. 11: 169. 1966. Leaf samples of *A. paihengii* were collected from Henan Agricultural University (Henan, China, 113°67′E, 34°79′N) and the specimens were deposited in the Herbarium of Henan Agricultural University (http://bbg.henau.edu.cn/, Liu Yiping and E-mail: Lyp_163@163.com) under the voucher number YJ20210325. Total genomic DNA was extracted using the OMEGA kit, after which an Illumina opposite-end library was constructed by Shanghai Yuanshen Biomedical Technology Co., Ltd. (Origingene, Shanghai, China) on an Illumina HiSeq TM sequencer (Illumina, San Diego, CA, USA). The raw data was approximately 5.26 G and low-quality sequences were filtered out to obtain clean and high-quality data. The chloroplast genome was assembled using the NOVOPlasty4.2 software (Nicolas et al. 2017). Gene annotation was performed using the PGA annotation software (https://github.com/quxiaojian/PGA) (Qu et al. 2019).

The whole length of the *A. paihengii* chloroplast genome (GenBank accession: MZ934750) is 155,967 bp, including a pair of 26,063 bp inverted repeat regions (IRa and IRb), a large single-copy (LSC) 85,798 bp region, and a small single-copy (SSC) 18,043 bp region. Furthermore, the total GC content of this circular DNA molecule was 35.62%. A total of 130 functional genes were annotated, including 85 protein-coding genes, 37 tRNA genes, and 8 rRNA genes. These three types of genes accounted for 65.39, 28.46, and 6.15% of all annotated functional genes, respectively. Among them, a total of 15 genes (*trnK*-UUU, *trnG*, *trnL*-UAA, *trnV*-UAC, *trnl*-GUA, *trnA*-UGC, *rps16*, *ropC1*, *atpF*, *petB*, *petD*, *rpl16*, *rpl2*, *ndhA*, and *ndhB*) contained one intron. In contrast, *clpP* and *rps12* possess two introns. Among which *trnI*-GUA, *trnA*-UGC, *rpl2*, *ndhB*, and *rps12* exist as double copies.

To study the phylogenetic position of *A. paihengii*, 22 complete chloroplast genome sequences were downloaded from the NCBI GenBank. Sequence alignment was performed using MAFFT v7.158b (Katoh and Standley 2013). A phylogenetic tree was then generated via maximum likelihood analysis in RaxML (Stamatakis 2014). The development analysis results indicated that most nodes in the phylogenetic tree were strongly supported and all 23 *Acer* plants were clustered in an evolutionary branch. *A. paihengii*, *A. coriaceifolium*, and *A. sino-oblongum* clustered together, indicating a close evolutionary relationship (Figure 1). In summary, the complete chloroplast genome of *A. paihengii* obtained in this study provides a robust basis for future phylogenetic studies of the *Acer* genus.

**Figure 1.**
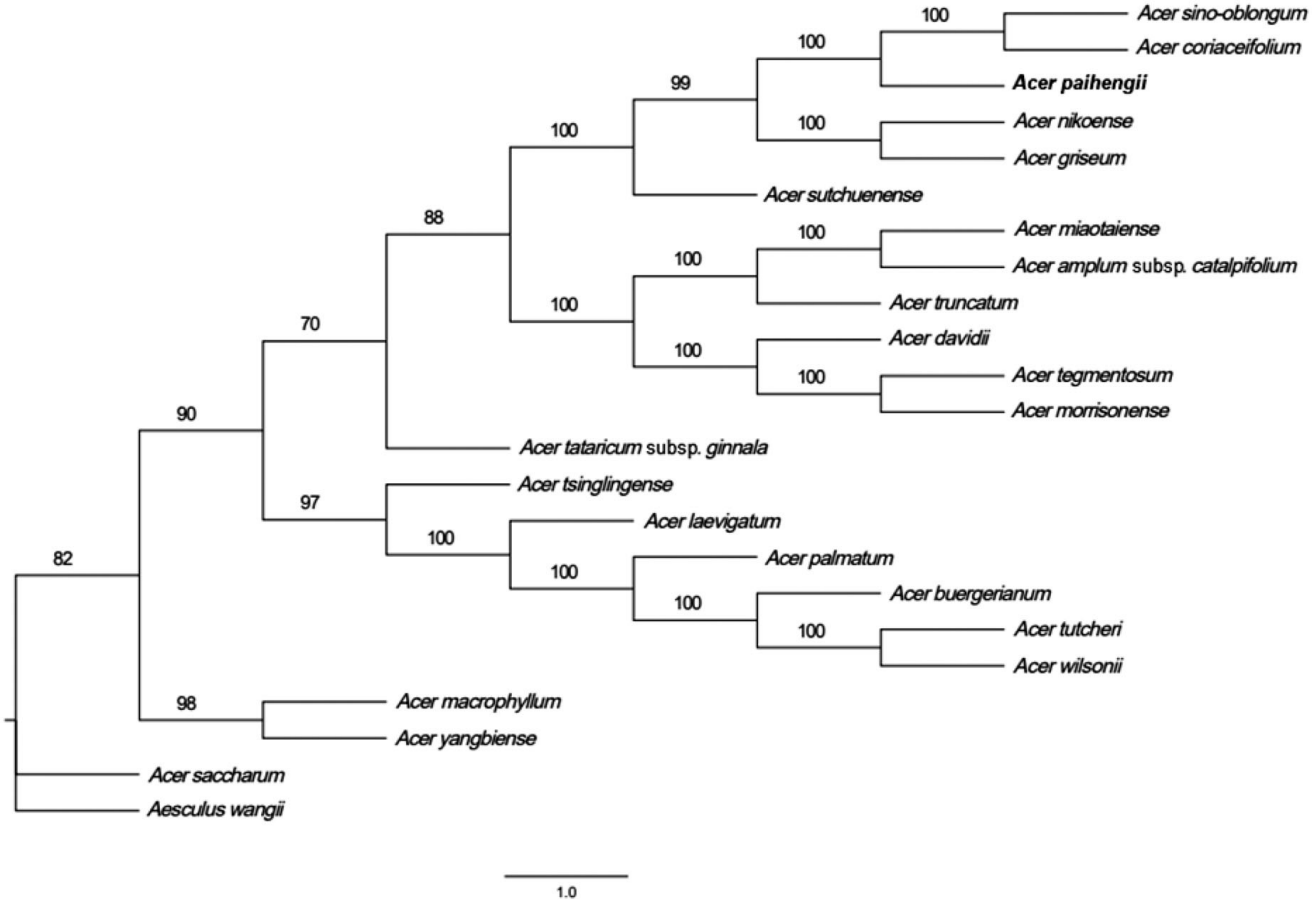
Phylogenetic tree of 23 complete chloroplast genome sequences of members of the order Sapindales. The numbers next to the nodes represent the bootstrap support values.

The following sequences were used: *Acer truncatum* NC_037211.1(Chen et al. 2019), *Acer miaotaiense* NC_030343.1 (Zhang et al. 2016), *Acer amplum* subsp. *catalpifolium* NC_041080.1 (Wang et al. 2019), *Acer tataricum* subsp. *ginnala* MN790641.1 (Yang et al. 2020), *Acer wilsonii* NC_040988.1, *Acer tutcheri* NC_051542.1 (Shi et al. 2020), *Acer buergerianum* NC_034744.1 (Xu et al. 2017), *Acer palmatum* NC_034932.1, *Acer laevigatum* NC_042443.1, *Acer tsinglingense* MN393475.1 (Dong et al. 2019), *Acer yangbiense* MN652924.1 (Ling and Zhang 2020), *Acer macrophyllum* NC_056217.1, *Acer saccharum* NC_051960.1 (Deng et al. 2019), *Acer sutchuenense* NC_049166.1 (Zhang et al. 2020), *Acer paihengii* MZ934750.1, *Acer sino-oblongum* NC_040106.1, *Acer cinnamomifolium* MN414240.1 (Chen et al. 2019), *Acer nikoense* NC_049165.1 (Fu et al. 2020), *Acer griseum* NC_034346.1 (Wang et al. 2017), *Acer davidii* NC_030331.1 (Jia et al. 2016), *Acer morrisonense* NC_029371.1 (Li et al. 2017), *Acer tegmentosum* MK942342.1 (Kim et al. 2019), *Aesculus wangii* NC_035955.1 (Zheng et al. 2018).

## Data Availability

The genome sequence data supporting the findings of this study are openly available in the NCBI GenBank database at https://www.ncbi.nlm.nih.gov/ under the accession no. MZ934750. The associated BioProject, SRA, and Bio-Sample numbers are PRJNA767933, SRR16970252, and SAMN22253490, respectively.
